# Glycolysis: The Bridge Between Cellular Interaction and Alzheimer’s Disease

**DOI:** 10.3390/biom16060796

**Published:** 2026-05-28

**Authors:** Qian Lei, Yinghan He, Bo Fang

**Affiliations:** Department of Anesthesiology, The First Hospital of China Medical University, NO. 155, North Nanjing Street, Heping District, Shenyang 110001, China; stu2024120864@cmu.edu.cn (Q.L.); 2021328516@cmu.edu.cn (Y.H.)

**Keywords:** glycolysis, cellular interactions, Alzheimer’s disease (AD), exosomes, tunneling nanotubes (TNTs)

## Abstract

The pathological progression of Alzheimer’s disease (AD) involves not only intrinsic neuronal lesions but is also closely associated with dysregulation of intercellular communication within the neuroimmune microenvironment. Glycolysis, as a central pathway in cellular energy metabolism, exhibits significant abnormalities in AD, and changes in its activity may further influence disease progression by modulating interactions between neurons. This article aims to systematically elucidate how glycolysis, as a key component of metabolic regulation, participates in the regulation of cellular interactions during the progression of AD, and to explore its potential mechanisms and therapeutic implications. Firstly, it systematically reviews the key changes in interactions between cells in AD, including microglia, astrocytes, oligodendrocytes, and neurons, and their roles in neuroinflammation, synaptic loss, and amyloid deposition. Secondly, by analysing alterations in the glycolytic metabolic profiles of various neuronal cell types in AD, we explore in depth how glycolysis regulates cellular signalling, inflammatory responses, and cellular function, thereby influencing cell–cell interactions. Lastly, by combining current research on the control of the glycolytic pathway in AD with possible therapeutic methods, we propose a novel way to stop the progression of the disease by focusing on cell interactions through mediating glycolysis. By tracing the causal chain in AD through which glycolysis acts as a bridge via altered cell–cell interactions, this paper aims to provide a theoretical basis for the development of disease-modifying therapies based on metabolic reprogramming.

## 1. Introduction

Alzheimer’s disease (AD) is a neurodegenerative condition marked by progressive cognitive decline, including deficits in executive function, attention, linguistic expression, and spatial visual processing [1,2]. The primary pathological mechanism is the progressive death or degeneration of neurons in the central nervous system (CNS) or peripheral nervous system (PNS) [3,4]. Its pathological hallmarks include extracellular β-amyloid plaques (Aβ) and intracellular neurofibrillary tangles created by hyperphosphorylated Microtubule-associated protein Tau (Tau) [5]. The number of AD patients keeps rising as the population ages more quickly [6], placing a significant strain on families and society. By 2050, AD and related dementias alone are expected to cost the world economy trillions of dollars, with a predicted total of $16.9 trillion [7].

Different cell types make up organisms, and cellular interactions are essential to preserving organismal behavior and function [8], impacting both development and maintaining homeostasis [9,10]. According to research, cellular communication regulates both the start and course of AD [11,12,13,14,15,16,17]. Through secretion and other processes, neurons and microglia engage in non-direct interactions in AD. Signaling pathways are disrupted and their strength is changed when intercellular ligands or receptors are expressed differently [12,14]. In addition to non-contact interactions, cells can move organelles or macromolecules by direct touch, such as through tunneling nanotubes (TNTs) [15,17]. Moreover, astrocytes control the density of their glial networks to modify the geographical separations from microglia, which restricts direct contact and modifies microglial activity [14].

Through the glycolytic pathway, a portion of the glucose used by the nervous system provides energy, intermediary metabolites, and other chemicals. By controlling a variety of cellular phenotypes, such as neuronal energy crisis, cellular maturity, apoptosis, mitochondrial function, oxidative stress, or inflammatory phenotypes, altered glycolytic flux affects the progression of AD [18,19,20,21,22,23,24]. Furthermore, glycolysis plays a role in regulating various intercellular interactions in AD [25,26,27].

Microglia, astrocytes, and neurons all rely on glycolysis, and their asynchronous reprogramming makes glycolysis a natural integration point for multicellular coordination [28]. Furthermore, glycolytic flux simultaneously drives biological processes such as energy metabolism, histone lactylation, inflammatory responses, reactive oxygen species (ROS) metabolism, exosome biogenesis, and lactate shuttling [29,30,31,32,33,34,35,36,37]. Changes in glycolytic flux determine whether intercellular interactions are in a state of compensatory coupling or pathological decoupling, thereby influencing the progression of AD [38,39]. This review defines glycolysis as a bridge for intercellular communication in AD. By analysing the regulatory mechanisms of glycolysis in cellular communication within AD, it proposes a view of stage-dependent glycolytic reprogramming in AD. Furthermore, this review summarises current potential therapeutic strategies for AD targeting glycolysis or cellular communication.

## 2. Cellular Interactions and Alterations in Glycolysis in AD

### 2.1. Cellular Interactions in AD

**Figure 1:**
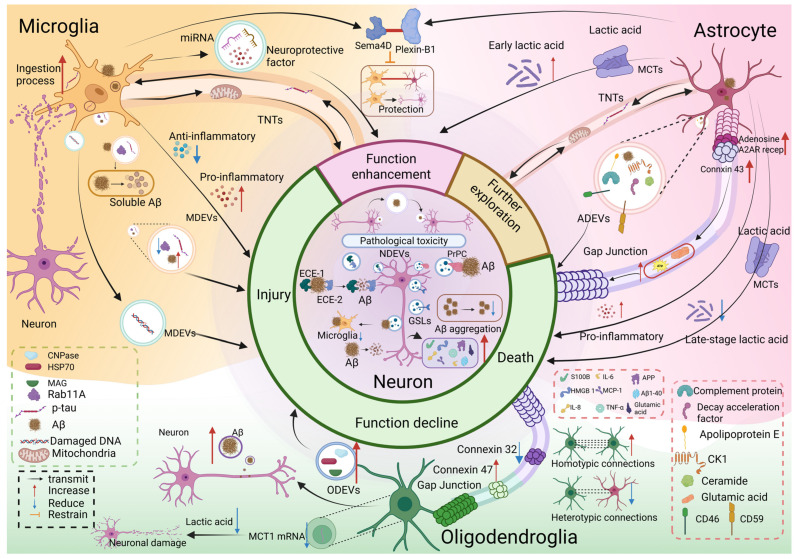
Altered cellular interactions in Alzheimer’s disease. Although the pathogenesis of Alzheimer’s disease centers on neuronal dysfunction and death, its progression is fundamentally regulated by intricate interactions between neurons and various cells within the neuroinflammatory microenvironment. Neurons do not exist in isolation, and their survival and function are highly dependent on a cellular network comprising the neurons themselves, as well as microglia, astrocytes, and oligodendrocytes. This pathological state is characterised by an imbalance in lactate secretion, a disruption of the inflammatory balance, abnormal transport of EVs, impaired substance transport via GJs and TNTs, reduced capacity for the phagocytic clearance of pathological proteins, and a decline in the ability to maintain myelin structure and function. These interactive abnormalities collectively accelerate neurodegenerative processes. Furthermore, alterations in cell–cell interactions extend beyond neurons. Changes in the spatial proximity between microglia and astrocytes directly impact the phagocytic efficiency of microglia toward amyloid plaques, thereby further regulating the pathological progression of Alzheimer’s disease.

In general, there are two types of intercellular interactions: direct contact interactions (TNTs, gap junctions (GJs), phagocytosis/contact-dependent interactions) and non-contact interactions (soluble secreted factors, extracellular vesicles (EVs) [40,41,42]. Cellular metabolic variables, especially glycolytic flux, control both of these cell–cell contact modalities, which are significantly altered in AD (Figure 1).

#### 2.1.1. Non-Direct Interactions

##### Soluble Secretory Factors

Cells secrete soluble substances (such as cytokines, chemokines, neurotransmitters, growth factors, and lactate) that interact with receptors on the surface of target cells in the most traditional paradigm of intercellular communication [40]. The profile of inflammatory factors secreted by neurons, astrocytes, and microglia changes significantly in AD: neuronal secretion of S100B, High mobility group box protein-1 (HMGB-1), Monocyte chemoattractant protein-1 (MCP-1), Interleukin-6 (IL-6), Interleukin-8 (IL-8), and Tumour necrosis factor-α (TNF-α) increases [43,44], astrocytes secrete more pro-inflammatory factors like TNF-α, Interleukin-1β (IL-1β), IL-8, and MCP-1, while microglia secrete more pro-inflammatory mediators like IL-1β, Interleukin-18 (IL-18), IL-6, TNF-α, inducible Nitric Oxide Synthase (iNOS), and Cyclooxygenase-2 (COX-2), while anti-inflammatory cytokines like Interleukin-10 (IL-10) [45,46,47].

In addition to inflammatory factors, increased secretion of Amyloid Precursor Protein (APP), Aβ1-40, and glutamate by neurons elevates the likelihood of neural damage [43,48]. Also, astrocytes affect AD in addition to inflammatory factors via modifying the astrocyte-neuron lactate shuttle (ANLS) through changes in lactate secretion. The process by which astrocytes absorb glutamate and initiate glycolysis after an increase in extracellular glutamate levels brought on by neuronal activity is known as the ANLS mechanism. Lactate is then released to neurons via monocarboxylate transporters (MCTs) to supply an energy substrate for neuronal oxidative metabolism [49,50,51]. Lactate shuttling is changed and shows clear stage-specific variations in AD, which affects the course of the disease [26,27,52]. Additionally, oligodendrocyte glycolysis is suppressed [53] and messenger ribonucleic acid (mRNA) of monocarboxylate transporter 1 (MCT1) expression levels are decreased in AD [54]. These results further imply that AD affects oligodendrocytes’ ability to support neurons’ lactate transfer.

##### Extracellular Vesicles (EVs)

Cells secrete EVs in addition to soluble substances that affect how they interact with each other. Additionally, exosomes, microvesicles, apoptosomes, and oncosomes are subtypes of EVs, which are nanoscale particles having a cell membrane structure [55]. Moreover, EVs are essential for intercellular communication and are involved in both physiological and pathological processes by transporting cell-specific bioactive substances like mRNA, non-coding RNA (ncRNA), proteins, and lipids [55]. In AD, EVs derived from various cell sources are involved in the propagation of pathology, the regulation of inflammation, alterations in myelin-related factors, and the regulation of neuroprotection [56,57,58,59].

Neuron-derived extracellular vesicles (NDEVs): They may operate as pathogenic proteins’ “scavengers” in addition to being their “transmitters”. Functionally specialized synaptic protein levels in plasma neuronal exosomes decrease as AD progresses [60], which may explain the early impairment of excitatory synaptic activity and subsequent cognitive loss in AD. Additionally, NDEVs may contribute to the spread of pathogenic proteins by acting as carriers for Aβ and active Tau protein [61,62,63,64]. Moreover, Aβ proteins can be captured by NDEVs, and introducing these neuronal exosomes into the brains of APP transgenic mice decreases Aβ and amyloid plaques [65,66]. NDEVs contain endothelin-converting enzyme-1 (ECE-1) and endothelin-converting enzyme-2 (ECE-2), which can degrade Aβ [67], glycosphingolipids (GSLs) on the membrane of NDEVs can drive the formation of amyloid protofibrils, thereby reducing Aβ aggregation [68], and the surface protein prion protein (PrPC) on NDEVs can bind directly to Aβ proteins [66]. Additionally, bound Aβ proteins can be transported by NDEVs to microglia, where they can be cleared [68].

Although the causes of these seemingly incompatible findings have not yet been investigated, they could be connected to the disease’s stage, the neurons’ metabolic condition, and the contents and membrane surface proteins of the EVs that are formed from those neurons.

Microglia-derived extracellular vesicles (MDEVs): Control neuronal activity and Aβ elimination. Aβ and Tau proteins are phagocytosed and internalized by microglia, which then release EVs carrying Aβ or phosphorylated Tau proteins. Additionally, the lipids in the EVs themselves facilitate the generation of soluble Aβ, which has a deleterious effect on neurons [69,70,71,72,73,74]. Microglia-derived EVs not only affect AD-associated pathogenic proteins, but these pathological molecules also change the makeup of the contents of microglia-derived EVs, which in turn affects a number of biological processes that microglia mediate. When microglia are exposed to Aβ, the amount of Ras-related protein 11A (Rab11A) protein in the EVs they produce decreases, which makes it harder for them to control Guanosine diphosphate/Guanosine triphosphate (GDP/GTP), which lowers the effectiveness of amyloid clearance and ultimately causes neuronal death [75]. Moreover, double-stranded deoxyribonucleic acid (dsDNA) can be encapsulated in EVs by microglial cells that have experienced deoxyribonucleic acid (DNA) damage and released into IFN-responsive neurons, causing neuronal death [76]. MDEVs, which secrete neuroprotective factors like Insulin-like growth factor 2 (IGF2), Integral membrane protein 2B (ITM2B), Cysteine aspartate protease 1 (CASPR-1), and DJ-1, as well as neuroprotective microRNAs (miRNAs) like miR-188-5p and miR-381-3p, also show a protective aspect in the brain tissue of AD patients [58]. The effects of microglia-derived exosomes are clearly two-sided. The precise underlying processes have not yet been investigated, although these opposing effects may be connected to extracellular vesicle subtypes or the stage of microglial activation [57,58].

Astrocyte-derived extracellular vesicles (ADEVs): Increasing neuroinflammation and controlling the metabolism and spread of Aβ proteins. Apolipoprotein E (APOE) and APP, the two most significant genetic risk factors for AD, and their ligand-receptor pairings are critical in the central nervous system (CNS), especially in the interactions between neurons and astrocytes. APOE is mostly released by astrocytes [77], and it plays a role in the control of lipid transport, synaptic function, and neuroinflammation [78] by attaching to certain receptors on neurons and microglia. By secreting EVs carrying APOE and Casein Kinase 1 (CK1), astrocytes affect the transcription, translation, and hydrolysis of APP in neurons, controlling the generation of Aβ [79,80,81,82]. Additionally, ADEVs may serve as direct carriers of Aβ disease. Astrocytes can release ADEVs with N-terminally shortened Aβ after phagocytosing Aβ, which causes neuronal death [83]. At the same time, Aβ itself can cause astrocytes to release ceramide-rich ADEVs, which reduces neurons’ ability to respond to energy metabolism [84]. Additionally, complement proteins (C1q, C4b, C3d, factor B, factor D, fragment Bb, C3b, and the C5b-C9 terminal complement complex), as well as the decay-accelerating factor (DAF), CD46, CD59, and complement receptor type I, can be transported by ADEVs, which increases neurotoxic inflammatory responses [47,85].

Oligodendrocyte-derived extracellular vesicles (ODEVs): They affect axonal function by controlling myelin assembly and deconstruction. EVs generated by oligodendrocytes are essential for preserving the axon’s structural and functional integrity because they can transport metabolic products, protective proteins, glycolytic enzymes, mRNA, and miRNA to particular locations along the axon [86]. By delivering antioxidants like superoxide dismutase and catalase and concurrently conveying autophagy-related proteins to control neuronal autophagy levels, these vesicles can also improve neuronal resilience to oxidative stress under pathological settings [87,88]. Oligodendrocyte exosomes are mainly secreted into the periaxonal space, but they can also be internalized by neuronal somata/dendrites, and axons through endocytosis [86,89]. Heat shock protein 70 (HSP70), myelin-associated glycoprotein (MAG), and 2′,3′-cyclic nucleotide 3′-phosphodiesterase (CNPase) levels in AD imply that oligodendrocyte-derived exosomes are involved in AD [90].

In conclusion, glial cells not only affect the metabolism of pathological proteins but also have an impact on the development of AD by controlling inflammatory reactions and myelin health, while NDEVs mainly mediate the distribution of toxic proteins in AD. This could be explained by variations in the intrinsic activities of different cell types, how they react to pathogenic signals, the different contents that EVs carry, and the intricate communication that occurs between them.

#### 2.1.2. Direct Interaction

In addition to non-direct interactions, cells can also exchange substances and information through contact-dependent mechanisms [91,92,93]. Indeed, these contact-dependent cellular interactions are altered in AD and influence the progression of the disease [14,15,94,95,96,97,98,99].

##### Tunneling Nanotubes (TNTs)

TNTs are a significant structure for intercellular communication that was just identified. TNTs are thin, hollow cell membrane protrusions that can create direct channels of intracellular communication between neighboring cells. These channels are essential for cellular control because they allow materials like proteins, vesicles, and organelles to move directly across cells [91]. As distinct extensions of the cell, TNTs enable bidirectional exchange of cellular contents between cells via direct physical connections, including the transfer of macromolecular structures like mitochondria, in contrast to the non-contact delivery mechanism of EVs [100,101].

TNTs serve two purposes in AD: first, they can carry toxic Tau aggregates from neurons to microglia, which facilitates the spread of pathology. Second, they can carry healthy mitochondria from microglia back to neurons, protecting the neurons [15,94]. TNTs can also facilitate Tau protein transmission and intercellular mitochondrial translocation in mixed cultures of neurons and astrocytes [102].

##### Gap Junctions (GJs)

A unique kind of transmembrane channel structure, GJs are made up of two opposing hemichannels connecting neighboring plasma membranes, each of which has six connexin subunits [95,103,104]. GJs facilitate quick metabolic coupling and electrical signal transmission by allowing ions and small-molecule metabolites (including glucose, lactate, adenosine triphosphate (ATP), glutamate, cyclic adenosine monophosphate (cAMP), etc.) to move directly between adjacent cells [92,93].

Astrocytes are essential for neuronal energy metabolism and synaptic activity because they carry glucose and lactate to neurons through connexin channels under healthy settings [93,105]. Gap junction-associated protein connexin 43 (Cx43), which interferes with intercellular communication via GJs and thus interferes with the metabolic support given to neurons, is also upregulated in astrocytes that proliferate in AD [95,96,103,106]. Elevated expression levels of the adenosine A2A receptor (A2AR) and Cx43, which aid in gap junction formation and in astrocytes in the pathological context of AD, encourage the aberrant release of neuroactive substances like ATP and glutamate, upsetting the metabolic balance of the brain microenvironment and resulting in neuronal death [95,96]. Moreover, under certain circumstances, gap junction activation can be blocked and neuronal damage mitigated by employing Cx43 inhibitors or knocking out Cx43 in astrocytes [107,108].

Oligodendrocyte precursor cells (OPCs) proliferate significantly in mice with an early-onset AD mouse model (5xFAD), and the expression of the junctional protein Cx47 is specifically upregulated in OPCs, while the expression of Cx32 is specifically downregulated [109]. This results in a restructuring of the gap junction architecture between the two types of glial cells, with an increase in homotypic connections between oligodendrocytes and a corresponding decrease in heterotypic connections between oligodendrocytes and astrocytes [109]. The glial network’s typical balance of metabolic coupling and signal transduction is effectively upset by this change in connection patterns, which has an immediate impact on oligodendrocyte functional homeostasis [109]. In the end, this affects neurons by significantly impairing their capacity to perform myelin repair and preserve myelin structural integrity [109].

##### Contact-Dependent Interactions

Phagocytosis

Direct cell–cell interactions contribute to the process of phagocytosis in addition to creating direct transport routes between cells. One important method by which microglia remove Aβ, apoptotic cells, and synaptic debris is phagocytosis. Microglia’s phagocytic function is significantly altered in AD. On the one hand, microglia can phagocytose and degrade Aβ proteins, which lessens the harmful effects of Aβ on neurons [97]. On the other hand, aberrant activation of microglia may cause dysfunction in their synaptic pruning function, which can lead to excessive synaptic clearance and even neuronal damage [98]. Microglia depletion can dramatically lower synaptic loss and neuronal degeneration [98], but it also removes microglia’s protective roles, indicating the need for careful modulation rather than total suppression.

Additionally, by adjusting the density of their glial network, astrocytes control the geographical distance between themselves and microglia, preventing direct contact and controlling microglial activity [14]. Sema4D-Plexin-B1 signaling pathway anomalies in AD impede this contact-dependent regulation mechanism [14]. Plexin-B1 belongs to the family of plexin axon-guiding receptors. Plexin-B1 expression is increased in astrocytes surrounding plaques in AD and it binds to Semaphorin-4D (Sema4D) secreted by nearby microglia, causing a cell-repulsion response that increases the distance between glial cells [14]. This inhibits microglial clearance of plaques and the neuroprotective effects of microglia on neurons, in addition to limiting communication between astrocytes and microglia.

Oligodendrocyte–axon structural support

Oligodendrocytes’ supporting role and the myelin sheaths they produce are severely compromised in AD. Oligodendrocytes serve as the structural and functional support units for neuronal axons. They do this by tightly encasing the axons in a multilayered lipid membrane called the myelin sheath, which not only greatly increases the speed of action potential conduction but also continuously supports the axons’ metabolism (e.g., by delivering glucose and lactate to the neurons) [110,111,112,113]. Damage to oligodendrocytes under pathological circumstances directly prevents myelination and compromises axonal integrity. It is important to note that myelin dysfunction and demyelination in AD might exacerbate Aβ plaque deposition and impair neuronal function by further inducing Aβ synthesis within axons and promoting aberrant cleavage of the APP [99]. Given its critical role in neuroprotection, the oligodendrocyte-myelin axis may be a key target for interfering with the pathological progression of AD. Research has demonstrated that myelin regeneration can effectively reverse cognitive impairment in AD mice [114].

### 2.2. Cell Type-Specific Changes in Glycolytic Flux in AD

**Figure 2:**
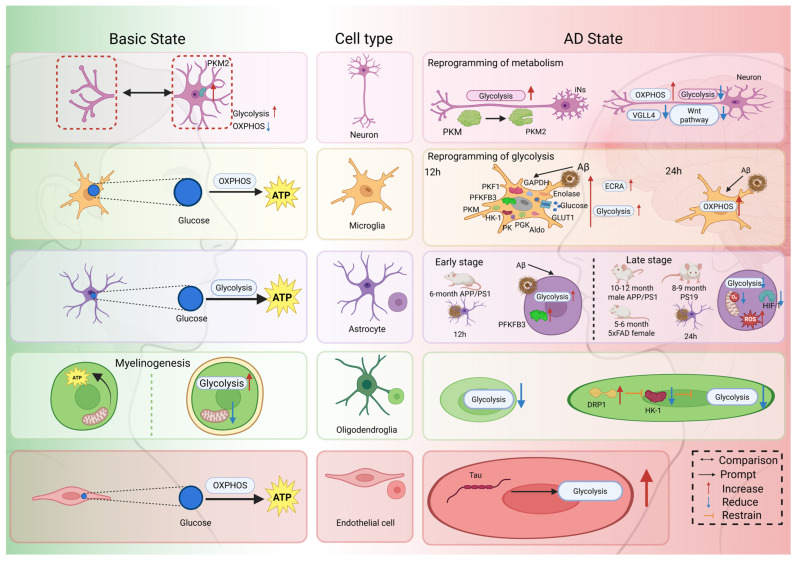
In Alzheimer’s disease, glycolysis exhibits significant and heterogeneous alterations in neurons, microglia, astrocytes, oligodendrocytes, and cerebral vascular endothelial cells. Among these, oligodendrocytes show a trend toward reduced glycolytic activity, while endothelial cells generally demonstrate enhanced glycolysis. In contrast, metabolic alterations in neurons, microglia, and astrocytes exhibit dynamic and context-dependent characteristics. Specifically, induced neurons (iNs) derived from Alzheimer’s disease patient fibroblasts tend to enhance glycolysis, whereas primary neurons are more likely to show increased dependence on oxidative phosphorylation (OXPHOS). The metabolic phenotypes of microglia and astrocytes are closely linked to disease progression or the duration of pathological stimuli: for example, short-term Aβ exposure drives microglia toward glycolysis, whereas prolonged stimulation shifts their metabolic preference toward OXPHOS. Similarly, astrocytes often exhibit upregulation of glycolysis in the early stages of disease to support rapid energy demands and neuroprotective functions, and as the disease progresses into later stages, their glycolytic activity tends to decline.

Glycolysis is a basic metabolic process that produces two pyruvate molecules from one glucose molecule to power cellular activity [115]. The brain relies heavily on glucose for energy, and as early as the preclinical stage of AD, there is a noticeable decrease in cerebral glucose metabolism. The normal physiology of brain cells is significantly impacted by functional alterations in glycolysis, a crucial part of glucose metabolism [116]. In many different types of cells in the CNS, glycolysis is essential. In the development and advancement of AD, glycolysis not only governs cellular energy supply, but its metabolic intermediates also participate in various pathophysiological processes, hence influencing the progression of AD at the energy and metabolic levels (Figure 2).

#### 2.2.1. Neurons

According to conventional wisdom, neurons mostly use mitochondrial oxidative phosphorylation (OXPHOS) to produce ATP under basal conditions, with glycolysis acting as the primary energy pathway mainly during neuronal activity and increased energy needs [31]. But new research shows that glycolysis has a long-term functional significance in brain metabolism that goes beyond activation phases. Research demonstrates that human neurons use glycolysis to metabolize glucose, providing substrates for the tricarboxylic acid (TCA) cycle [117]. In mice, neuronal dysfunction is directly induced by knocking out neuronal glucose transporters or neural-specific pyruvate kinase (PK) subtypes to block glycolysis. This results in age-related decreases in learning and memory capacity [117]. Interestingly, differences in neuronal metabolic results may be related to spatial compartmentalization within neurons rather than just being the result of experimental error or different observation time points. Different neuronal compartments have different metabolic preferences. For example, Pyruvate kinase muscle isozyme 2 (PKM2), a crucial glycolytic regulator, is mostly found in the soma rather than axon terminals. As a result, in both basal and activated states, neuronal somata exhibit lower OXPHOS levels and higher aerobic glycolysis than axon terminals [31].

Research results on neuronal glycolysis in AD are likewise contradictory. Studies reveal increased glycolysis and Warburg-like metabolic traits in induced neurons (iNs) derived from AD patient fibroblasts, including a change in PKM to PKM2 and a metabolic shift from primarily OXPHOS to aerobic glycolysis [23]. Nevertheless, a number of investigations have also suggested that malfunctioning neurons in AD may display an opposing metabolic pattern, marked by increased OXPHOS without a discernible elevation in glycolysis [118,119,120,121]. This opposite alteration may be linked to the suppression of the Wnt pathway and the downregulation of neuronal Vestigial-like family member 4 (VGLL4) [119,121]. VGLL4 downregulation can result in decreased sensitivity of neuronal lactate dehydrogenase A (LDHA) to hypoxia, which in turn inhibits glycolysis [121].

Since research supporting enhanced glycolysis in AD has primarily focused on induced neuronal iNs models, which may have limitations in accurately reflecting the complex metabolic interactions observed in vivo, as they fail to fully account for the regulatory influence of other neuronal cell types and the external environment on neuronal metabolism. It can be surmised that the discrepancies between the aforementioned research findings may stem from insufficient consideration of factors other than the neurons themselves (particularly components of the microenvironment). Nevertheless, the glycolytic characteristics of neurons produced from various neuronal subtypes, culture conditions, or differentiation conditions, as well as their responses to Aβ/Tau activation, have not yet been directly compared in an analogous experimental setup. One of the main reasons the current disputes within the area are hard to resolve is the absence of a systematic comparison.

#### 2.2.2. Microglia

Microglia mostly use OXPHOS to produce ATP when they are at rest. On the other hand, the metabolic state of microglia is highly dynamic, especially in pathological situations like AD [33,122]. Research has demonstrated that microglia in the hippocampus of AD models have reduced OXPHOS performance along with increased glucose absorption and glycolysis [123]. Enzymes related to glycolysis, including PKM, hexokinase-1 (HK-1), and phosphoenolpyruvate carboxylase 3 (PFKFB3), have markedly increased expression levels in aged microglia [124]. Proteomic research also shows that exposure to AD patient plasma enhances the expression of several glycolytic enzymes in microglia, such as PK, phosphoglycerate kinase (PGK), enolase, aldolase, and glyceraldehyde-3-phosphate dehydrogenase (GAPDH) [125]. Further research indicates that glycolytic enzymes such as phosphofructokinase 1 (PFK1) and PFKFB3 are activated in microglia under AD circumstances [32,33,48,126]. By increasing the expression of the glucose transporter type 1 (GLUT1), microglia also improve glucose absorption and glycolysis in inflammatory settings, in addition to changes in enzymes related to glycolysis [127]. An important connection between the two main pathogenic processes of aging and neuroinflammation in AD is this metabolic change in microglia from OXPHOS to aerobic glycolysis [29,32,128,129].

Interestingly, glycolytic reprogramming in AD microglia shows dynamic adaptive alterations rather than a straightforward unidirectional improvement. According to available data, the response may vary depending on the stage of the disease and the duration of stimulation. According to studies, microglia display increased glycolysis and extracellular acidification rate (ECAR) after 12 h of exposure to Aβ, although this changes to enhanced OXPHOS after 24 h [130]. This time-dependent metabolic change could be the result of microglia switching from an acute compensatory response to a long-term adaptation. Additionally, bioinformatic investigations show that as AD advances, glycolysis is downregulated in a number of different brain areas, and that this downregulation is tightly linked to the activation state of microglia [129]. Therefore, determining the neurotoxic or neuroprotective roles of microglia in AD requires a methodical examination of variations in their metabolic state throughout the disease’s stages and within various brain microenvironments.

#### 2.2.3. Astrocytes

According to earlier research, astrocytes mostly rely on glycolysis rather than OXPHOS to meet their bioenergetic needs while at rest [131]. Astrocytes undergo substantial metabolic remodeling in AD; however, the research that is now available seems to contradict this. While some studies show glycolytic failure under Aβ/Tau pathological activation [131,132,133], others suggest the increase in glycolysis in response to an energy shortage [27,48,134,135,136,137]. A duality in metabolic reprogramming may be shown by this discrepancy between early or adaptive compensatory augmentation and late or pathological functional decline. Understanding the changing involvement of astrocytes in disease requires clarifying the transition points and mechanisms.

AD astrocytes have increased glycolysis, according to several studies [27,48,134,135,136,137]. Astrocytes showed increased glycolysis and raised L-lactate levels after 12 h of Aβ administration. However, this impact was no longer significant after 24 h, indicating a temporary elevation of glycolysis in the early stages of the disease [27]. Systemic metabolic reprogramming is induced by APOE4, the biggest genetic risk factor for late-onset AD. Research verifies that when lactate is present, APOE4-expressing astrocytes show increased glycolytic activity, decreased oxygen consumption, and decreased glucose oxidation rates, which suggest increased aerobic glycolysis (the Warburg effect) [136]. During acute stress, this metabolic change might be protective. The idea of enhanced glycolysis in AD pathogenesis is further supported by the notably heightened activity of reactive astrocytes and the glycolytic enzyme PFKFB3 around Aβ plaques [137].

However, additional research indicates that AD inhibits astrocyte glycolysis [131,132,133,138]. Improved bioenergetic function, as evidenced by decreased glycolysis, decreased mitochondrial oxygen consumption, and enhanced ROS generation, was found by proteomic analysis of immortalized hippocampal astrocytes from 3xTg-AD animals [132]. Moreover, it has been demonstrated that Aβ suppresses the production of hypoxia-inducible factor 1 (HIF-1), which lowers the rate of glycolysis and encourages aberrant astrocyte activation [133].

Similar to microglia, variations in astrocyte glycolysis reprogramming may be linked to AD stage [26,27]. Increased astrocyte glycolysis plays a compensatory function in the early stages of AD, but persistent imbalance alters the lactate-glucose metabolic coupling, which ultimately exacerbates the neurodegenerative processes and neuronal energy crises typical of AD [26,27]. Nevertheless, no research has yet dynamically monitored changes in glycolysis in a single experimental system from early to late stages in the same cohort of astrocytes. Additionally, the majority of studies use non-physiological high concentrations of Aβ (e.g., 10–20 μM), which may avoid the compensatory phase and cause toxic effects directly. As a result, the process of enhanced astrocyte glycolysis is not observed [139,140].

#### 2.2.4. Oligodendrocytes and Endothelial Cells

Oligodendrocytes in the brain use a significant amount of energy to synthesize and maintain myelin [141]. These cells mostly rely on mitochondrial respiration to produce ATP before myelin formation. But after myelinization is finished, they become less reliant on mitochondrial respiration as their metabolic preference changes to glycolysis [141,142]. This metabolic change may be caused by the fact that OXPHOS is better at keeping OPCs in their undifferentiated state, which ensures their survival and proliferation, while the intermediates of glycolysis give mature oligodendrocytes the precursors for myelin lipid and protein synthesis and cause mature oligodendrocytes to produce fewer mitochondrial ROS (mROS), which lowers oxidative DNA damage [112,143]. Additionally, mature oligodendrocytes under stress have limited glycolysis. This could be because oligodendrocytes purposefully decrease glycolysis to increase OXPHOS to guarantee ATP synthesis [144].

Oligodendrocyte glycolysis is also impaired in AD. Significant failure in the oligodendrocyte glycolytic pathway is revealed by post-mortem RNA sequencing investigations of AD patient brain tissue, underscoring its dysregulation in the AD clinical process [53]. Due to excessive activation of dynamin-related protein 1 (DRP1), suppression of oligodendrocyte glycolysis may be linked to inhibition of HK-1 [145]. Additionally, research shows that blocking oligodendrocyte glycolysis causes inflammasome activation, which in turn causes axonal degeneration and demyelination in AD patients [145]. Furthermore, a form of glycolytic regulation of the interaction between oligodendrocytes and neurons may be represented by the significant impairment of the glycolytic pathway in oligodendrocytes in AD, which may lead to impaired metabolic support for neurons through lactate transfer by oligodendrocytes [53,54]. Additionally, studies on multiple sclerosis (MS) show that utilizing metformin to increase glycolysis in OPCs can speed up oligodendrocyte development in both healthy and myelin-damaged animals, hence increasing myelin repair [146].

Blood–brain barrier (BBB) impairment is intimately linked to AD, according to research [147,148,149,150]. Cerebral microvascular endothelial cells, the anatomical and functional basis of the BBB, are essential to the development and course of AD [150]. Through mechanisms such as instability in the endothelial microtubule cytoskeleton, activation of endothelial Nitric Oxide Synthase (eNOS), aberrant actin organization, and oxidative stress, Aβ, APP, Tau, and other factors can cause endothelial cell senescence or even death [150,151,152,153]. Cerebral endothelial cells contribute to the generation of Aβ in addition to these pathogenic protein effects. According to research, brain endothelial cells can create soluble Aβ by glycosylating APP770, which promotes AD pathogenesis. Additionally, the AD state modifies the metabolism of endothelial cells. Tau protein increases endothelial cells’ glycolytic activity and encourages them to adopt a pro-inflammatory phenotype, which results in aberrant cell adhesion molecule production and localization and eventually jeopardizes the integrity of the BBB [154].

## 3. Glycolysis Regulates Cellular Interactions

A number of variables, including the microenvironment, extracellular signals, metabolic status, and epigenetic regulation, affect intercellular interactions. These intercellular processes are also heavily influenced by and controlled by glycolysis, a key element of cellular energy metabolism. One of the main pathogenic pathways causing neurodegeneration and synaptic dysfunction in AD is dysregulation of cell–cell connections mediated by anomalies in glycolysis (Table 1).

### 3.1. Glycolysis–Lactate Coupling

Glycolysis is the central pathway for lactate production, and the two are closely coupled in terms of metabolic flux, redox balance, and intercellular energy transfer. In recent years, a growing body of research has shown that lactate is not merely a metabolic by-product of glycolysis. It also serves as an energy carrier, a substrate for epigenetic modification, and a signalling molecule. Its functions exhibit a high degree of duality in both physiological and pathological contexts, and it plays a significant role in cell–cell interactions in AD [49,112,164,165].

On the one hand, extracellular acidity is impacted by the pathological build-up of excess lactate in AD, which impairs cellular function. According to research, Aβ causes microglia to increase glycolysis and lactate production, which releases too much lactate into the cellular milieu and damages neurons [155]. On the other hand, by altering histone lactylation, lactate generated by glycolysis can potentially disrupt cell–cell interactions. According to recent studies, excess lactate can enter microglia’s nuclei and cause histone H4 at lysine 12 (H4K12) and histone H3 at lysine 18 (H3K18) to become more lactylated [29,35].

Additionally, through changes in ANLS, the interaction between astrocytes and neurons also helps to regulate glycolysis in disease. Research has demonstrated that Aβ-induced reactive astrocytes have decreased glucose uptake and impaired glycolysis, which results in decreased lactate release and poor neuronal energy metabolism [131,138]. Interventions like blocking the function of the mitochondrial pyruvate carrier (MPC) in astrocytes, encouraging the production of lactate from glycolysis, or directly injecting L-lactate intraperitoneally in AD model mice. Thereby, increasing the supply of lactate to neurons has been demonstrated to lower the levels of Tau and amyloid aggregates in the brains of AD animal models, improve synaptic plasticity, and restore cognitive function in response to the attenuation of glycolysis in astrocytes [37,166]. Relevant research indicates that lactate production in astrocytes may be linked to the advancement of AD, even if investigations examining the relationship between astrocyte lactate metabolism and disease development have not yet been carried out. In the early stages of AD (6-month-old APP/PS1 mice or astrocytes treated with Aβ oligomers for 12 h), glycolysis in astrocytes is enhanced, and lactate secretion increases to compensate for neuronal energy demands [27]. In contrast, in the late stages (10–12-month-old male APP/PS1 mice, 5–6-month-old female 5xFAD mice, and 8–9-month-old PS19 mice, or astrocytes treated with Aβ oligomers for 24 h), glycolysis is inhibited, resulting in neuronal metabolic imbalance and functional decline [26,27]. However, earlier research has also shown that 6-month-old APP/PS1 animals already have impaired astrocyte glycolysis [52]. The reasons for this disparity are yet unknown. Possible interactions between astrocytes and microglia, or the representativeness of the age of AD model mice for the disease stage, could be significant factors [27,52]. In conclusion, astrocyte glycolysis and ANLS in AD are extremely dynamic and complex. Understanding their regulation mechanisms is crucial for investigating metabolic intervention techniques for AD.

Additionally, ANLS maintains nitrogen homeostasis in the milieu of the CNS by mediating glutamate uptake and connecting it to the glutamate-glutamine cycle between astrocytes and neurons [167]. Through high-affinity transporters, astrocytes effectively absorb glutamate generated by neurons and, with the help of glutamine synthetase, transform it into the inert form glutamine. In addition to promoting neurotransmitter renewal and taking part in energy metabolism and antioxidant production, glutamine returns to neurons as a safe carrier of carbon and nitrogen. However, in the initial phases of neuroinflammation, astrocytes’ enhanced glycolysis and increased S100B gene expression and release prevent them from absorbing glutamate. Although the actual connection between increased glycolysis and reduced glutamate uptake has not yet been established, this may result in increased oxidative stress in neurons, leading to neuronal injury [32]. Astrocytes with APP or presenilin-1 (PSEN-1) mutations show decreased expression of excitatory amino acid transporter 2 (EAAT2) in cellular models that mimic AD, resulting in a reduced ability to absorb glutamate [48]. Increased glutamate release is also seen in neurons with PSEN-1 mutations, which together cause glutamate buildup. Additionally, astrocytes and neurons with APP or PSEN-1 mutations show increased glycolysis and glucose oxidation when compared to wild-type (WT) controls [48]. These results imply that mutations linked to familial AD (fAD) cause astrocytes to exhibit a hypermetabolic phenotype, which may then encourage the build-up of glutamate toxicity and neuronal damage [48].

To preserve axonal function, oligodendrocytes can use the MCT1 channel on their cell membrane to deliver lactate from astrocytes or themselves to neurons [112,168]. Research has demonstrated that AD impairs oligodendrocyte glycolysis, which lowers lactate generation. Axonal degeneration results from oligodendrocytes lacking MCT1, which not only produce less lactate but also transport less lactate and pyruvate to axons [54,156].

### 3.2. Glycolysis-Inflammation Coupling

In addition to influencing the production of metabolic by-products, glycolysis can also influence neuroinflammation, thereby affecting AD. Through the activation of the NF-κB pathway [157] and the NLRP3 inflammasome [32,145], the reprogramming of glycolysis in glial cells in the cerebral environment of AD causes an accumulation of inflammatory factors and metabolically toxic products, which exacerbates cognitive impairment and promotes the death of neurons and glial cells.

Microglia are quickly activated by acute exposure to Aβ, which results in increased glycolysis and the release of several pro-inflammatory mediators, such as TNF-α, IL-1β, IL-6, IL-8, and Nitric Oxide Synthase 2 (NOS2) [20,29,34,35,126,169,170]. The reprogramming of glycolysis in astrocytes can affect the expression of pro-inflammatory genes, such as complement component 3, IL-1β, IL-6, and TNF-α, at both the mRNA and protein levels, thereby affecting the regulatory role of astrocytes in neuroinflammation [171], as well as the secretion of the paracrine molecule S100B, thereby affecting microglial-mediated neuroinflammation [32,158]. On the other hand, changes in oligodendrocyte glycolysis can cause autologous inflammation and pyroptosis, which can result in myelin loss and white matter degeneration. This weakens the protective function of myelin on neurons and exacerbates neuronal damage [145].

Although there is currently little research on glycolysis-mediated inflammation in AD, what is known indicates that activation of the NLRP3 inflammasome and the NF-κB pathway may be implicated. The rate-limiting enzyme of glycolysis in microglia, hexokinase-2 (HK-2), is markedly upregulated in AD. However, mild suppression of HK-2 expression can raise the levels of its cytoplasmic target, IKBα (NF-κB inhibitor α), which reduces NF-κB’s nuclear translocation and the transcription of genes linked to inflammation [157]. Additionally, inhibition of glycolysis in microglia (e.g., 3-(3-pyridinyl)-1-(4-pyridinyl)-2-propen-1-one (3PO) and oxalamide, which inhibit the glycolysis-related enzymes PFKFB3 and lactate dehydrogenase (LDH)) can attenuate NLRP3 inflammasome assembly in microglia, thereby reducing the cleavage of the IL-1β precursor into its active form and reducing neurotoxicity [32]. In contrast, inhibition of mature oligodendrocyte pyroptosis and demyelination [145]. These two cell types regulate the NLRP3 inflammasome differently through glycolysis, and research has not yet confirmed the mechanisms underlying these differences. This, however, might be connected to the cells’ reliance on glycolysis: under physiological conditions, mature oligodendrocytes rely more on glycolytic function, while microglia tend to rely more on OXPHOS [33,122,141,142].

Nevertheless, glycolysis does not always control the release of inflammatory cytokines. In comparison to microglia treated with acute Aβ, glycolysis in microglia is significantly reduced in the late stages of Aβ stimulation (24 h after treatment of microglia with oAβ, followed by washing off the oAβ and further culture for 3–5 days), and the release of pro-inflammatory cytokines is correspondingly diminished [20].

### 3.3. Glycolysis-Oxidative Stress Coupling

Furthermore, glycolysis is a key metabolic process for preserving cellular redox homeostasis because it reduces mitochondrial ROS production and increases nicotinamide adenine dinucleotide phosphate (NADPH)-mediated antioxidant activity through the metabolism of methylglyoxal (MGO), creating a dynamic equilibrium with oxidative stress [31,172,173,174].

Aβ treatment of microglia increases the activity of the pentose phosphate pathway (PPP) and the glycolytic enzyme glucose-6-phosphate dehydrogenase (G6PD), which activates nicotinamide adenine dinucleotide phosphate oxidase (NOX) and ultimately increases the production of ROS, which directly damages neurons [159]. In AD, astrocytes’ expression of glycolytic enzymes is inhibited, which lowers their glycolytic rate and causes them to switch to OXPHOS-dependent metabolism, which increases ROS generation and oxidative stress [24]. On the other hand, glucagon-like peptide-1 (GLP-1) analogues, like liraglutide, shield neurons by encouraging astrocyte glycolysis and preventing the generation of ROS [24].

Glycolysis reprogramming is also impacted by oxidative stress. Glycolysis-related enzymes in affected brain regions have all undergone oxidative modification, according to redox proteomic analysis of AD brain tissue [175]. This could be a major contributing factor to the decreased glucose metabolism and consequent drop in ATP production seen in the brains of AD patients.

### 3.4. Glycolysis-Exosomes Coupling

Aggregates of Tau and Aβ proteins can be transported by exosomes from neurons to nearby microglia [176,177]. Aβ or Tau proteins can also cause glycolytic reprogramming in glial cells [178].

The soluble precursor peptides of Aβ plaques, amyloid β protein oligomers (AβOs), can stimulate the expression of MCT1 and its co-receptor CD147 in microglia, mediating glycolytic reprogramming and increasing exosome production in microglia [36]. In the end, these exosomes make AD more neurotoxic [36].

### 3.5. Glycolysis-Phagocytosis Coupling

One important way that the brain uses glucose metabolism to supply energy for cellular activity is through glycolysis [115,116]. It is common knowledge that energy is necessary for the body to operate normally, and changes in glycolytic flow in AD have a regulatory effect on cellular connections by altering the energy supply.

Under physiological conditions, microglia continue to be in a state of dynamic surveillance. They collectively maintain the homeostasis of the neural microenvironment by performing crucial functions such as mediating synaptic pruning to optimize neural circuits and constantly patrolling the microenvironment to remove aberrant protein aggregates and apoptotic cells. Increased glycolytic activity in AD causes microglia’s phagocytic capacity to decrease, which exacerbates neuronal damage by accumulating Aβ. According to current research, glycolysis is less energy-efficient than OXPHOS and finds it difficult to meet the high energy requirements of phagocytosis [33,159]. Consequently, it is plausible to hypothesize that the decreased Aβ phagocytosis in microglia may be associated with an inadequate energy supply [22,33,159,160,161,162]. It is important to remember that increased microglial phagocytosis may cause harm to neurons or their connections. Therefore, boosting microglial phagocytosis indiscriminately may potentially be harmful to the advancement of AD.

### 3.6. The Interplay of the Above Mechanisms

#### 3.6.1. Glycolysis–Lactate–Inflammation Cross-Talk

Aβ can cause microglial metabolism to change from OXPHOS to glycolysis, which increases the formation of lactate. The change in lactate levels brought on by glycolysis not only directly affects the cellular microenvironment and neuronal energy metabolism, but it also modifies the lactylation of histones, which in turn affects the expression of inflammatory markers. Glycolysis can be further enhanced by histone lactylation, creating a positive feedback loop known as “glycolysis–histone lactylation–inflammation” [29,35]. Research has demonstrated that increased lactate secretion and enhanced glycolysis in AD microglia cause increased H4K12 lactylation (H4K12la). This lactate-dependent histone modification is then enriched in the promoter regions of glycolytic genes (like Hif-1, PKM, and LDHA), which in turn causes further enhancement of glycolysis, which in turn causes microglial proliferation and increased secretion of inflammatory factors [29]. By increased binding to the promoters of Rela (p65) and NF-κB1 (p50), elevated levels of H3K18 lactylation (H3K18la) directly trigger the NF-κB signaling pathway, upregulating the production of IL-6 and IL-8 in microglia and augmenting neuroinflammation [35].

#### 3.6.2. Glycolysis–Lactate–Exosome Cross-Talk

Lactic acid can also influence the release of exosomes by affecting the electrolyte balance in the cytoplasm. Research on how depressive stress affects AD has revealed that, in the context of AD, depressive stress enhances the activation of glycolysis in microglia, producing huge levels of lactate. Through a non-lactate-dependent mechanism, this rise in lactate can increase the activity of the microglial potassium channel Kv1.3, lowering intracellular potassium contents in microglia. This accelerates the onset of cognitive decline by promoting the release of Aβ-laden exosomes into the extracellular space, which exacerbates the distribution of dangerous Aβ proteins within the brain [163].

## 4. Prospective Treatment

Targeting glycolysis has emerged as a possible technique for interfering with the pathological course of AD, as glycolysis plays a role in this process [179]. However, precise staging boundaries are still difficult to establish since glycolysis plays different functional roles in different types of neuronal cells and its effects change dynamically over the course of a disease. As a result, indiscriminate glycolysis increase or inhibition may have negative consequences or possibly worsen pathological damage. Research on glycolysis modulation as a treatment for AD is now in its experimental stage. Given that not all current studies targeting glycolysis for the treatment of AD have progressed to clinical trials, and some therapeutic strategies have only been validated in preclinical animal studies, it is not possible to directly apply the GRADE grading system [180] to evaluate the various therapeutic strategies. We therefore combined the ARRIVE guidelines (Animal Research: Reporting of In Vivo Experiments) [181] and SYRCLE’s RoB tool [182] to classify existing therapeutic strategies as follows: Level 1: randomised controlled trials (RCTs) or meta-analyses based on RCTs, Level 2: Phase I/II clinical trials, Level 3: AD transgenic animal model studies, Level 4: non-AD animal model studies (e.g., ageing models, other neurodegenerative disease models), Level 5: in vitro cell experiments. Currently, research into treating AD by modulating glycolysis remains at an exploratory stage, and studies with evidence levels 1–2 are still rare (Table 2).

### 4.1. Strategies for Inhibiting Glycolysis

#### 4.1.1. Purine/Pyrimidine Compounds

Purines and pyrimidines are essential components of nitrogenous bases. Adenine, guanine, hypoxanthine, and xanthine are the main six- and five-membered nitrogenous heterocycles that make up purines [183]. Uracil, thymine, and cytosine are the main examples of pyrimidines, which are composed of a single six-membered nitrogenous ring [183]. Purine and pyrimidine chemicals may contribute to the regulation and therapy of AD by directly inhibiting enzymes or by creating kinase inhibitors, according to several studies [184,185,186]. And many kinases are involved in the key regulation of glycolysis, such as HK-2, PFK1, and PKM2 [115]. Studies have demonstrated that N6-isopentenyladenosine (iPA) reduces PKM2 expression by inhibiting the NF-κB kinase subunit β/NF-κB pathway inhibitor, thereby suppressing glycolysis in glioblastoma [187]. Nevertheless, there are currently no transgenic AD animal models or clinical trials, and the effects of these medicines have only been confirmed in AD animal models injected with Aβ protein. Further research is needed to determine whether these substances can be utilized for therapeutic treatment because they are precursor molecules for DNA synthesis, long-term usage of both may pose a risk of genotoxicity, and the glycolytic pathway currently lacks specific targeting capabilities.

#### 4.1.2. Uncarin

Uncarin (UR), the main active component of Uncaria rhynchophylla (Chinese: Gou Teng), a traditional medicinal plant in the Rubiaceae family, has been shown to be an important therapeutic agent for the treatment of AD [188,189]. According to research, Uncaria’s indole alkaloids have positive effects on AD [188,190]. These effects may be due to the suppression of glycolysis, which inhibits CD4 T cell-mediated neuroinflammation [191]. The pathogenic aspects of AD, such as improved cognitive performance, decreased tau phosphorylation and Aβ accumulation, and decreased neuronal death, have been shown to be significantly improved by treatment with alkaloid compounds from Uncaria (URA) [191]. Additionally, neuroinflammation is significantly reduced, as seen by higher levels of TGF-β and IL-10 and decreased levels of IFN-γ and IL-17 [191]. However, research using animal models of AD is the only source of evidence that Uncarin enhances cognitive performance in AD by blocking glycolysis, and its exact mode of action is yet unknown [191]. Furthermore, Uncarin’s anti-inflammatory and cognitive-enhancing effects may not be only dependent on the regulation of glycolysis because its positive effects on AD involve multi-target mechanisms [192,193]. Given these drawbacks, more clinical validation and mechanistic studies are still needed to fully understand the potential therapeutic efficacy of Uncarin, an active ingredient in traditional Chinese medicine, in treating AD.

### 4.2. Strategies for Promoting Glycolysis

#### 4.2.1. α-Adrenergic Receptor Antagonist

α-adrenergic receptor antagonists have been considered as medications with potential therapeutic efficacy since the hippocampal and prefrontal cortex of AD patients exhibit noradrenergic impairment [194,195,196]. Further research suggests that by encouraging glycolysis, these substances may help cure neurodevelopmental problems [179,197,198,199]. As α-adrenergic receptor antagonists, terazosin, doxazosin, and alfuzosin (Tz/Dz/Az) can bind to and activate important ATP-generating enzymes in glycolysis, including the glycolytic enzyme PGK1, thereby promoting glycolysis and slowing the progression of ND [179,197,198,199,200,201]. Terazosin (Tz) has been shown to have dose-dependent effects on glycolysis, according to recent research [200]. While high-concentration Tz may competitively inhibit PGK1 activity, low-concentration Tz binding to PGK1 reroutes metabolism into bypass pathways, speeding product release and increasing enzymatic phosphotransferase rates [200]. As a result, in clinical applications, dose control needs special consideration. It is important to note that while α-adrenergic receptor antagonists have shown neuroprotective effects against AD in vitro in hippocampal tissue and in AD transgenic mice [194,195,196], the field of Parkinson’s disease (PD) is the primary source of proof-of-concept for treating neurodegenerative diseases by increasing glycolysis. Although specific investigations aimed at AD have not yet been carried out, this offers a ready-made mechanistic entry point and an urgent subject for interdisciplinary investigation in AD research.

#### 4.2.2. IDO1 Inhibitor

In AD research, Aβ causes tau pathology and neurotoxicity by downregulating the Wnt/β-catenin signaling pathway and upregulating the indoleamine 2,3-dioxygenase (IDO1)-kynurenine (Kyn)-aromatic hydrocarbon receptor (AhR) signaling system. IDO1 inhibitors, however, can counteract these effects [202,203,204,205,206,207,208]. The natural product tryptophan anthraquinone and its derivatives, along with 1-aryl-1H-naphtho [2,3-d][1,2,3]triazole-4,9-dione derivatives, a miconazole analogue, and the traditional Chinese medicinal formula Oren-gedoku-to (OGT), are among the many IDO1 inhibitors that have been discovered recently and have all shown promise in the treatment of neurodegenerative diseases [204,205,206,207,208]. IDO1 inhibitors may work through glycolysis, according to recent studies [26]. By encouraging astrocytic glycolysis, boosting astrocytic lactate generation, and improving neuronal lactate uptake, the selective IDO1 inhibitor PF06840003 (PF068) slows the degenerative course of AD [26]. IDO1 inhibitors have not produced favorable clinical results in clinical trials for tumor immunotherapy, according to a number of tumor studies, and their safety profile is still being assessed [209,210]. In conclusion, the risk of clinical translation is still significant even though the mechanism by which it controls glial-neuronal metabolic coupling appears appealing.

#### 4.2.3. GLP-1 Receptor Agonists

The metabolism of glucose is intimately linked to GLP-1, and new research suggests that GLP-1 and its receptor agonists may be useful in treating ND [211,212,213]. One way that GLP-1 may work is through glycolysis. GLP-1 activates the PI3K/Akt pathway, mediating a metabolic shift in astrocytes from OXPHOS to aerobic glycolysis. This improves synaptic function, sustains neuronal survival, increases axonal and dendritic development, strengthens astrocytic support for neurons, and lessens AD symptoms [24]. GLP-1 and its receptor agonists, including exenatide, liraglutide, and semaglutide, are therefore thought to have therapeutic potential for AD [214]. Liraglutide is safe and well-tolerated in individuals with mild to moderate AD, although its cognitive-enhancing effects on AD are not significant, according to a new clinical trial [215]. The regulating effects of GLP-1 and its receptor agonists on AD cannot be completely ruled out because this study only looked at GLP-1 receptor agonists and evaluated a single dose.

#### 4.2.4. Cannabidiol (CBD)

The main non-psychoactive phytochemical that is derived from cannabis leaves is cannabidiol (CBD). With a steady rise in pertinent research in recent years, interest in its potential for treating AD has grown due to its role in preventing oxidative stress, lowering inflammation, reducing pathological protein deposits, and encouraging the production of neuroprotective factors [216,217,218,219]. The potential of CBD to modulate alterations in glycolysis may be intimately linked to these effects. According to these studies, CBD significantly lowers levels of pro-inflammatory cytokines TNF-α and IL-6, increases glycolytic capacity and glycolytic reserve in astrocytes in a cannabinoid receptor type 1 (CB1)-dependent manner, and reduces ROS production in LPS-stimulated astrocytes, all of which have therapeutic effects in AD models [220]. While preclinical trials have confirmed the significance of CBD in improving cognitive function in AD [221], investigations on astrocytes have so far only examined its protective effects mediated by glycolysis [220]. Further research is needed to determine the mechanism by which CBD controls AD and whether it targets a particular cell type, because changes in glycolysis among various cell types in AD are not synchronized.

#### 4.2.5. Probiotics

The microbiota–gut–brain axis is one way that the gut microbiota may affect CNS processes, according to a growing body of studies in recent years [222]. According to a recent study, oral probiotics improve glucose absorption and promote glycolysis by restoring the glucose transporter type 3 (GLUT3), GLUT1, and insulin-like growth factor receptor β expression levels in the brains of 3xTg-AD mice [223]. This implies that one important mode of action for probiotics may be the modulation of brain glycolysis, which calls for more research in this area.

#### 4.2.6. Ketones/Ketogenic Diet

Apart from oral probiotics, dietary supplements that contain ketone esters (KE), such as D-β-hydroxybutyrate and R-1,3-butanediol, can also help mice with AD-related metabolic abnormalities and sustain high glycolysis levels [224]. It is widely acknowledged that the ketogenic diet increases levels of metabolites in glycolysis and the TCA cycle, which helps prevent ND [224,225], while also supplying ketone bodies as an alternative energy source for the brain [226,227,228,229]. Oral KE, however, boosted acetyl-CoA and TCA cycle intermediates in non-fasting mice, which in turn inhibited glycolysis through feedback mechanisms, according to a recent study on acute neurological diseases [230]. This result seems to go against the previously mentioned research, indicating that in acute situations, the primary function of ketone body metabolism may be the direct inhibition of glycolysis by its direct product, acetyl-CoA. In chronic pathological conditions of AD, on the other hand, ketone bodies can function as an alternative fuel to support overall glucose metabolism. However, only animal studies in AD transgenic mice have been conducted on the beneficial effects of ketone bodies in AD, and the mechanism of action is still unknown [224]. It is not yet clear whether this effect results from compensatory regulation brought on by the feedback inhibition of glycolysis or from the direct action of the ketone bodies themselves.

### 4.3. Supplementary Metabolites Related to Glycolysis

#### L-Serine

The glycolytic intermediate 3-phosphoglycerate is the first step in the de novo synthesis of L-serine [235]. L-serine production is decreased when glycolysis in astrocytes is hindered in AD, which mediates the disease’s progression. On the other hand, oral L-serine supplementation helps prevent synaptic and behavioral abnormalities in AD animals [131]. This viewpoint has since been contested, though. Research shows that when AD pathology and symptoms worsen, PHGDH expression rises. This elevation may indicate increased serine synthesis because PHGDH expression is strongly associated with the synthesis of L-serine and its byproduct D-serine [231]. Furthermore, by reducing the conversion of L-serine to D-serine, knocking down serine racemase (SR) decreases the generation of D-serine while surprisingly reducing the neurotoxicity caused by injection of the Aβ_1–42_ peptide [232]. Remarkably, later research showed that altered glycolytic flow is the main cause of decreased L-serine synthesis in astrocytes, which does not always correlate with PHGDH expression levels [233]. Additionally, PHGDH shows sex-specific post-translational modification regulation in the AD brain. In particular, male AD patients show specific deacetylation of the K289 residue in PHGDH within hippocampal tissue, which results in decreased L-serine stability and further complicates this pathway [234]. The effectiveness of oral L-serine supplementation to boost D-serine levels and so rectify the pathological effects of glycolytic irregularities is still debatable, given the conflicting findings mentioned above and current studies. To clarify the function of L-serine in AD and to methodically assess its safety and effectiveness in treatment, more clinical studies or basic research are needed.

## 5. Conclusions

We think that a detailed examination of the particular regulatory mechanisms of glycolysis within the cellular interaction network of AD, as well as an explanation of the dynamic changes and cell-specific variations in glycolysis across various pathological stages of AD, will not only contribute to a better understanding of the mechanisms underlying the pathological progression of AD, but also offer a strong theoretical foundation for the creation of innovative intervention strategies that target the “metabolic-cellular feedback loop.” It is anticipated that extensive research in this multidisciplinary area will lead to a paradigm shift in AD treatment strategies in the future, shifting from conventional single-target interventions to multidimensional systemic regulation and providing fresh perspectives and innovations for the clinical management of AD. This study methodically integrates and analyzes changes in intercellular interactions between various cell types in AD, possible explanations for the contradictory results regarding cellular glycolysis in current research, and the precise mechanisms by which glycolysis controls intercellular interactions based on available research evidence. The “glycolysis–lactate–inflammation cross-talk” and the “glycolysis–lactate–exosome cross-talk” are the two regulatory paradigms it suggests. Based on the aforementioned research, we suggest the following important conclusion: targeted modulation of glycolytic metabolic pathways offers promise as a unique possible method for the treatment of AD since glycolysis serves as a critical intermediary bridge connecting intercellular interactions and AD.

However, current research on abnormalities in glycolytic metabolism and intercellular interactions in AD remains fraught with controversy and inconsistent findings. Given the limitations of the duration and depth of existing research, this review has systematically analysed the potential causes underlying some of these controversies, such as the functional heterogeneity of exosomes from different cellular sources and the possible reasons for differences in glycolytic abnormalities across cell types in AD. The review process has also revealed that there remain numerous scientific questions requiring further investigation, specifically including: precise classification criteria and quantification methods for the different pathological stages of AD, the underlying molecular mechanisms leading to specific glycolytic and functional abnormalities in different cell types, and the overall molecular networks and regulatory patterns through which glycolysis modulates intercellular interactions. However, the stage-specific and cell-type-specific alterations in glycolysis observed in existing studies suggest that establishing a unified model of how glycolysis influences disease progression would be of significant value to Alzheimer’s disease research. These unresolved key issues clearly identify the core research directions for future studies in the field of AD metabolism and cell–cell interactions.

Additionally, current research has demonstrated that there is substantial heterogeneity in glycolytic metabolic changes across various pathological stages and cell types in AD. Therefore, research in the following three areas is highly valuable from a scientific and clinical standpoint: (1) conducting long-term longitudinal studies to systematically monitor the dynamic evolution of glycolytic metabolism from early to late-stage AD within a unified experimental framework; (2) developing cell-type-specific metabolic detection technologies to enable real-time, accurate assessment of glycolytic flux in key cell types, including astrocytes, microglia, and neurons in vivo; and (3) designing clinical trials based on a stage-based framework of AD pathology to systematically investigate the safety and clinical efficacy of stage-dependent glycolytic intervention strategies. Only through the aforementioned research can ideal therapeutic strategies be identified that specifically target glycolysis at different pathological stages of AD or in specific cell types.

We think that a detailed examination of the particular regulatory mechanisms of glycolysis within the cellular interaction network of AD, as well as an explanation of the dynamic changes and cell-specific variations in glycolysis across various pathological stages of AD, will not only contribute to a better understanding of the mechanisms underlying the pathological progression of AD, but also offer a strong theoretical foundation for the creation of innovative intervention strategies that target the “metabolic-cellular feedback loop.” It is anticipated that extensive research in this multidisciplinary area will lead to a paradigm shift in AD treatment strategies in the future, shifting from conventional single-target interventions to multidimensional systemic regulation and providing fresh perspectives and innovations for the clinical management of AD.

## Figures and Tables

**Table 1:** Altered Cell Interactions and Underlying Mechanisms in Alzheimer’s Disease due to Glycolysis.

Functional Coupling Types	Cellular Interactions Involved	Glycolysis-Mediated Alterations	Potential Mechanism	References
Glycolysis–lactate coupling	Microglia–Cellular microenvironment–Neurons	Aβ stimulates microglia to release excess lactate into the extracellular microenvironment		[155]
	Astrocytes–Neurons	Reduced lactate release from astrocytes leads to a decrease in the metabolic substrates available to neurons		[131,138]
	Astrocytes–Neurons	Inhibition of glutamate uptake by astrocytes disrupts nitrogen homeostasis in the extracellular microenvironment	Promote the secretion of S100B	[32]
			Astrocytes exhibit reduced expression of excitatory amino acid transporter 2 (EAAT2)	[48]
	Oligodendrocytes–Neurons	Reduced levels of lactate, an energy substrate transported by oligodendrocytes to axons	Glycolysis is inhibited in oligodendrocytes, and there is a deficiency of monocarboxylate transporter 1 (MCT1) on their cell membranes	[54,156]
Glycolysis-inflammation coupling	Microglia–Neurons	Increased secretion of various pro-inflammatory mediators in microglia, including IL-1β, TNF-α, IL-6, IL-8, and Nitric Oxide Synthase 2 (NOS2)	Increased expression of hexokinase 2 (HK-2) promotes the nuclear translocation of NF-κB and enhances the transcription of inflammation-related genes	[157]
			Promotes the assembly of the NLRP3 inflammasome, leading to the cleavage of IL-1β into its active form	[32]
	Astrocytes–Neurons	Promotes the expression of pro-inflammatory genes in astrocytes, such as complement component 3, IL-1β, IL-6, and TNF-α	Promotes the secretion of the paracrine molecule S100B	[32,158]
	Oligodendrocytes–Neurons	Triggers autoinflammation and pyroptosis in oligodendrocytes, leading to myelin damage and white matter degeneration, thereby exacerbating neuronal damage	Promotes NLRP3 inflammasome activation	[145]
Glycolysis-oxidative stress coupling	Microglia–Neurons	Increased ROS production leads to greater neuronal damage	Increased levels of the glycolytic enzyme glucose-6-phosphate dehydrogenase (G6PD), enhanced activity of the pentose phosphate pathway (PPP), and activation of nicotinamide adenine dinucleotide phosphate oxidase (NOX)	[159]
	Astrocytes–Neurons	Increased ROS production leads to greater neuronal damage	The shift in glucose metabolism from glycolysis to oxidative phosphorylation (OXPHOS) leads to increased ROS production	[24]
Glycolysis-exosome coupling	Microglia–Neurons	Increased exosome secretion leads to enhanced neurotoxicity	Increased expression of monocarboxylate transporter 1 (MCT1) and its chaperone protein CD147 promotes exosome biogenesis and release	[36]
Glycolysis-Phagocytosis Coupling	Microglia–Cellular microenvironment–Neurons	Impairs Aβ clearance, leading to neuronal and synaptic damage	The low energy output of glycolysis is insufficient to meet the high energy demands of phagocytosis	[22,33,159,160,161,162]
Glycolysis–lactate–inflammation cross-talk	Microglia–Neurons	Increased histone lactylation and enhanced neuroinflammation in microglia	Increased H4K12 lactylation (H4K12la) further enhances glycolysis and promotes increased secretion of inflammatory cytokines by microglia	[29]
			Increased H3K18 lactylation (H3K18la) stimulates the NF-κB signalling pathway, leading to increased secretion of pro-inflammatory factors	[35]
Glycolysis–lactate–exosome cross-talk	Microglia–Neurons	Upregulation of glycolysis promotes the production of large amounts of lactate and increases the release of exosomes	Lactic acid upregulates Kv1.3 channel activity, reduces intracellular potassium concentrations, and promotes the release of Aβ-laden exosomes and the spread of toxicity	[163]

**Table 2:** Evidence Level and Limitations of Treatment Strategies.

Treatment Strategies	Highest Standard of Proof	Evidence Specific to AD	Main Limitations	References
Purine/pyrimidine compounds	Level 4 (research not involving AD animal models)	Yes	Genotoxicity risk, lack of validation of specific targets	[115,183,184,185,186,187]
Uncarin (UR)	Level 3 (AD animal model research)	Yes	Unclear mechanism, multiple targets	[188,189,190,191,192,193]
α-adrenergic receptor antagonist	Level 2 (Clinical Trials)	very few	The inference that Parkinson’s disease may lead to AD is invalid	[179,194,195,196,197,198,199,200,201]
IDO1 inhibitor	Level 3 (AD animal model research)	Yes	The clinical benefits and safety of the drug also remain to be assessed, and the risks associated with clinical translation are high	[26,202,203,204,205,206,207,208,209,210]
GLP-1 receptor agonists	Level 2 (Clinical Trials)	Yes (but negative)	Clinical RCTs failed to demonstrate therapeutic efficacy	[24,211,212,213,214,215]
Cannabidiol (CBD)	Level 3 (AD animal model research)	Yes	No clinical trials are available, and the pathological basis is confined to astrocytes	[216,217,218,219,220,221]
Probiotics	Level 3 (AD animal model research)	Yes	A lack of exploration into the underlying mechanisms	[222,223]
Ketones/Ketogenic diet	Level 3 (AD animal model research)	Yes	The relevant mechanisms remain unclear	[224,225,226,227,228,229,230]
L-serine	Level 3 (AD animal model research)	Yes	Contradictory evidence and questionable therapeutic efficacy	[131,231,232,233,234]

## Data Availability

No new data were created or analyzed in this study.

## Abbreviations

AD	Alzheimer’s disease
TNTs	Tunneling nanotubes
CNS	Central nervous system
PNS	Peripheral nervous system
Aβ	β-amyloid plaques
Tau	Microtubule-associated protein Tau
ROS	Reactive oxygen species
GJs	Gap junctions
EVs	Extracellular vesicles
HMGB-1	High mobility group box protein-1
MCP-1	Monocyte chemoattractant protein-1
IL-6	Interleukin-6
IL-8	Interleukin-8
TNF-α	Tumour necrosis factor-α
IL-1β	Interleukin-1β
IL-18	Interleukin-18
iNOS	Inducible Nitric Oxide Synthase
COX-2	Cyclooxygenase-2
IL-10	Interleukin-10
APP	Amyloid Precursor Protein
ANLS	Astrocyte-neuron lactate shuttle
MCTs	Monocarboxylate transporters
mRNA	Messenger ribonucleic acid
ncRNA	Non-coding RNA
NDEVs	Neuron-derived extracellular vesicles
ECE-1	Endothelin-converting enzyme-1
ECE-2	Endothelin-converting enzyme-2
GSLs	Glycosphingolipids
PrPC	Prion protein
MDEVs	Microglia-derived extracellular vesicles
Rab11A	Ras-related protein 11A
GDP	Guanosine diphosphate
GTP	Guanosine triphosphate
dsDNA	Double-stranded deoxyribonucleic acid
DNA	Deoxyribonucleic acid
IGF2	Insulin-like growth factor 2
ITM2B	Integral membrane protein 2B
CASPR-1	Cysteine aspartate protease 1
miRNAs	MicroRNAs
ADEVs	Astrocyte-derived extracellular vesicles
APOE	Apolipoprotein E
CK1	Casein Kinase 1
DAF	Decay-accelerating factor
ODEVs	Oligodendrocytes-derived extracellular vesicles
HSP70	Heat shock protein 70
MAG	Myelin-associated glycoprotein
CNPase	2′,3′-cyclic nucleotide 3′-phosphodiesterase
ATP	Adenosine triphosphate
cAMP	Cyclic adenosine monophosphate
Cx43	Connexin 43
A2AR	Adenosine A2A receptor
OPCs	Oligodendrocyte precursor cells
5xFAD	An early-onset AD mouse model
Sema4D	Semaphorin-4D
MCT1	Monocarboxylate transporter 1
OXPHOS	Oxidative phosphorylation
TCA	Tricarboxylic acid
PK	Pyruvate kinase
PKM2	Pyruvate kinase muscle isozyme 2
iNs	Induced neurons
VGLL4	Vestigial-like family member 4
LDHA	Lactate dehydrogenase A
HK-1	Hexokinase-1
PFKFB3	Phosphoenolpyruvate carboxylase 3
PGK	Phosphoglycerate kinase
GAPDH	Glyceraldehyde-3-phosphate dehydrogenase
PFK1	Phosphofructokinase 1
GLUT1	Glucose transporter type 1
ECAR	Extracellular acidification rate
HIF-1	Hypoxia-inducible factor 1
mROS	Mitochondrial ROS
DRP1	Dynamin-related protein 1
MS	Multiple sclerosis
BBB	Blood–brain barrier
eNOS	Endothelial Nitric Oxide Synthase
H4K12	Histone H4 at lysine 12
H3K18	Histone H3 at lysine 18
MPC	Mitochondrial pyruvate carrier
PSEN-1	Presenilin-1
EAAT2	Excitatory amino acid transporter 2
WT	Wild-type
fAD	Familial AD
NOS2	Nitric Oxide Synthase 2
HK-2	Hexokinase-2
3PO	3-(3-pyridinyl)-1-(4-pyridinyl)-2-propen-1-one
LDH	Lactate dehydrogenase
MGO	Methylglyoxal
PPP	Pentose phosphate pathway
G6PD	Glucose-6-phosphate dehydrogenase
NOX	Nicotinamide adenine dinucleotide phosphate oxidase
GLP-1	Glucagon-like peptide-1
AβOs	Amyloid β protein oligomers
H4K12la	H4K12 lactylation
H3K18la	H3K18 lactylation
RCTs	Randomised controlled trials
iPA	N6-isopentenyladenosine
UR	Uncarin
Tz	Terazosin
Dz	Doxazosin
Az	Alfuzosin
PD	Parkinson’s disease
IDO1	Indoleamine 2,3-dioxygenase
Kyn	Kynurenine
AhR	Aromatic hydrocarbon receptor
OGT	Oren-gedoku-to
CBD	Cannabidiol
CB1	Cannabinoid receptor type 1
GLUT3	Glucose transporter type 3
KE	Ketone esters
